# microeco 2: A comprehensive R package for downstream analysis of microbiome omics data

**DOI:** 10.1002/imt2.70132

**Published:** 2026-05-16

**Authors:** Chi Liu, Xiangzhen Li, Felipe R. P. Mansoldo, Tong Chen, Fanzheng Meng, Ruixiang Tang, Siyu Zhou, Qinghua Yang, Ruixin Shao, Minjie Yao

**Affiliations:** ^1^ Engineering Research Center of Soil Remediation of Fujian Province University; College of Resources and Environment, Fujian Agriculture and Forestry University Fuzhou China; ^2^ State Key Laboratory of High‐Efficiency Production of Wheat‐Maize Double Cropping/College of Agronomy Henan Agricultural University Zhengzhou China; ^3^ Universidade Federal do Rio de Janeiro, Instituto de Química, LAGOA‐LADETEC, Rio de Janeiro Rio de Janeiro Brazil; ^4^ State Key Laboratory for Quality Ensurance and Sustainable Use of Dao‐di Herbs, National Resource Center for Chinese Materia Medica China Academy of Chinese Medical Sciences Beijing China; ^5^ Key Laboratory of Bioresources and Ecoenvironment (Ministry of Education), Sichuan Key Laboratory of Conservation Biology on Endangered Wildlife, College of Life Sciences Sichuan University Chengdu China; ^6^ State Key Laboratory of Genetic Engineering, School of Life Sciences, Human Phenome Institute Fudan University Shanghai China

**Keywords:** data normalization, diversified methods, function refactoring, machine learning, metagenomics

## Abstract

Efficient downstream analysis of microbiome data remains a major challenge for researchers. Since its initial release in late 2020, the R microeco package has been widely used for downstream statistical analysis and visualization of omics data, such as amplicon sequencing. Compared with its initial release, the current second version of the microeco package has undergone extensive updates and enhancements. The key upgrades include: (1) The addition of classes for data normalization and machine learning, respectively; (2) The incorporation of additional analytical methods and the addition of functions across various classes; (3) Optimization of the parameter system to expand the applicable scenarios of relevant methods; (4) Code restructuring to enhance the connectivity between statistical analysis and visualization within each class; (5) Extension of certain functions to enable the analysis of abundance data in complex formats generated from bioinformatic analyses of metagenomic/metatranscriptomic data; (6) Incorporation of several analytical methods commonly used in transcriptomic and metabolomic data analyses. Overall, the microeco package 2.0 offers broader method coverage and a wider range of application scenarios compared to the previous version and other existing R packages. The steady growth in user downloads demonstrates that the microeco package, which is built on R6 (a class‐based object‐oriented programming system for R), has established a broad and active user base. The second version of the microeco R package is open‐source and available on the Comprehensive R Archive Network and GitHub (https://github.com/ChiLiubio/microeco).

## Highlights

- The second version of the microeco package integrates more method categories and algorithms.
- The code has been refactored for a more logically sound architecture.
- The package supports downstream analyses of complex metagenomic bioinformatics data.

## Graphical abstract

The current version 2.0 of the microeco package represents a substantial update compared to the previously published version. Key enhancements include the addition of new classes, integration of a broader suite of analytical methods, a comprehensive overhaul of the parameter system, improved connectivity between statistical analysis and visualization functions, enhanced capability to process complex‐format metagenomic bioinformatics data files, and support for multi‐omics data analysis.

## INTRODUCTION

The development and popularization of microbiome technologies, such as amplicon sequencing, have driven rapid advances in microbe‐related research across diverse fields [1]. Amplicon sequencing data analysis primarily involves two stages [2]. First, preliminary bioinformatics generates abundance and taxonomic profiles for amplicon sequence variants (ASVs) or operational taxonomic units (OTUs). Second, downstream analysis builds upon these datasets to derive biological insights. Downstream procedures, including various data preprocessing steps, statistical analyses, and visualization, are generally characterized by high methodological complexity. A wide array of tools is available for such analyses, which can be categorized into two types. The first type comprises cloud‐based platforms and standalone software accessible to users without prior programming experience, such as MicrobiomeAnalyst [3], ImageGP [4], and iNAP [5]. These tools are relatively user‐friendly, as the underlying analytical workflows are predefined by developers. The second type includes programming‐based analytical environments, such as the R language and its extension packages [2]. For instance, the vegan package is widely used for biodiversity analysis [6], while the ggClusterNet package is designed for microbial network analysis [7]. To address the complexity of microbiome data, several R packages have integrated diverse analytical approaches to streamline workflows and enhance efficiency. Representative examples include phyloseq [8], microeco [9], and MicrobiotaProcess [10]. In addition, to facilitate the utilization of different R packages and other analytical tools, a number of workflow repositories have emerged, such as EasyAmplicon [11] and MicrobiomeStatPlots [12]. We released the initial version of the microeco package on the Comprehensive R Archive Network (CRAN) at the end of 2020, aiming to streamline downstream statistical analysis and visualization of amplicon sequencing data [9]. This package employs a modular R6 class‐based design that offers greater flexibility than S4 class‐based frameworks.

As microbiology‐related research has advanced across diverse disciplines, integrated multi‐omics strategies have been increasingly adopted in scientific investigations. Among these omics approaches, the analysis of metagenomic/metatranscriptomic sequencing data presents the greatest complexity, as the output file formats generated by different bioinformatics software tools vary substantially [1]. The abundance data derived from metagenomic/metatranscriptomic data often differ in format from those produced by amplicon sequencing. Amplicon‐based analyses typically yield ASV/OTU abundance tables paired with taxonomic annotation files, which constitute the standard data format widely adopted in community ecology. In contrast, outputs from metagenomic pipelines frequently adopt non‐standardized structures. For instance, the abundance data of genes or metabolic pathways produced by the HUMAnN software [13] incorporates taxonomic information across diverse species. This necessitates flexible options for calculating the relative abundance or reads per kilobase (RPK) abundance of genes/metabolic pathways at subsequent analytical levels. Furthermore, the ontology annotations in the MetaCyc database [14], which underpin metabolic pathway analysis, may be associated with multiple labels at the superclass level. This also requires the calculation of metabolic pathway abundance to accommodate such complex data structures. Therefore, downstream R packages must be highly compatible to effectively process outputs from metagenomic sequencing pipelines. This presents several challenges to the design of parsing methods for the R package: the parsing approach should capture all necessary data information, supplement missing data when available, and enable flexible manipulation to accommodate downstream analyses under diverse conditions. For other omics data types, such as metabolomics and transcriptomics, it is likewise essential to integrate not only general‐purpose analytical approaches but also the conventional methods established through the history of technical development. Such integration is critical for enabling end users to implement unified and streamlined analytical workflows for multi‐omics data processing.

In the years following the publication of the initial version of the microeco package, we have implemented substantial upgrades to it. The current Version 2.0 integrates an expanded analytical suite and features a more sophisticated design logic. Ongoing maintenance of microeco focuses on multi‐omics data parsing, ensuring robust compatibility with various metagenomic bioinformatics outputs. Since its initial submission to the CRAN repository, the microeco package has experienced a steady increase in user adoption over the past 5 years, with peak monthly downloads exceeding 4000 and cumulative downloads surpassing 120,000 to date. Moreover, microeco has been applied in more than 1600 publications (Google Scholar by April 2026). This paper provides a comprehensive overview and systematic analysis of the major updates introduced in the second version of the microeco package.

## RESULTS

### Summary of major updates in the second version of the microeco package

**Figure 1:**
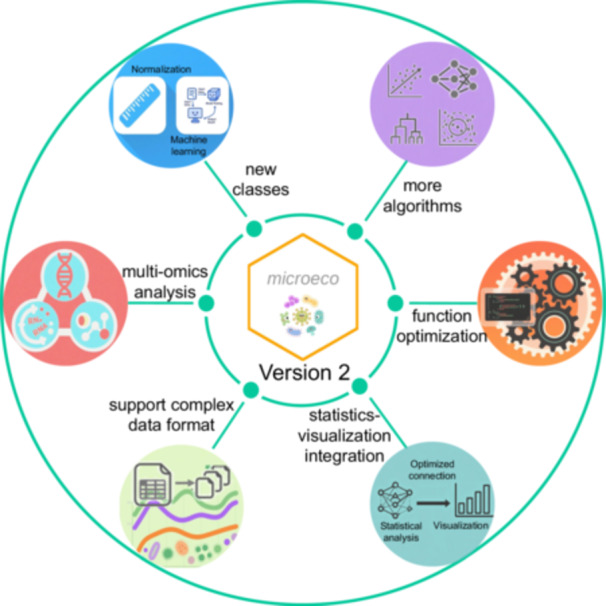
A summary diagram of the main upgraded contents of the second version of the microeco package.

**Figure 2:**
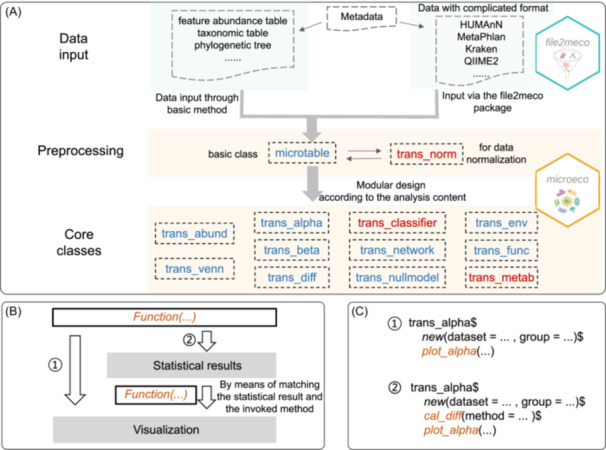
Design framework and logic of the second version of the microeco package. (A) The design framework of the microeco package version 2. Among them, the red text (trans_norm and trans_classifier) means they are newly added classes in the microeco package version 2. (B) The design logic of statistical and visualizing functions in core classes of the microeco package version 2. ① A method for direct visualization; ② An approach involving statistical analysis prior to visualization. “By means of matching the statistical result and the invoked method” means that the visualization function will automatically select an appropriate method for visualization based on the stored statistical analysis result data and the analysis method contained within the object. (C) The demonstration of the functions and the usage of the pipe operator ($) for the two methods described in (B), with the trans_alpha class as an example. Most visualization functions in each class are directly executable. For visualizations based on statistical analysis results, the relevant functions and default parameters will be automatically invoked to generate outputs for visualization purposes.

Compared with its initial release, the second version of the microeco package has undergone substantial updates in terms of analytical methodologies (Figure 1). Key updates encompass six major areas: (i) structural expansion via new classes like trans_norm and trans_classifier; (ii) algorithmic enrichment through the integration of more statistical approaches; (iii) systematic refinement of functions and parameters; (iv) seamless statistical analysis and visualization workflows; (v) support for the analysis of input data with complex formats; and (vi) integration of multi‐omics analytical approaches. The internal design logic of each class has also been refined based on more rational principles (Figure 2). In particular, remarkable improvements have been made to the calling of result data when combining statistical analysis with visualization, with strict adherence to the principle of separating statistical operations from visualization processes. The overall system has been made more user‐friendly through the renaming of certain functions, parameters, and analytical results within the classes. In addition, seamless integration with the file2meco package has facilitated the input of complex bioinformatics data outputs (e.g., metagenomic data) (Figure 2). Moreover, extensive details regarding methodological implementations have been added to the package documentation.

### Newly added classes

The newly added class trans_norm is specifically designed for normalizing input data. At its core function (*norm*), it integrates a wide range of normalization methods as well as data transformation techniques. Normalization methods applicable to amplicon sequencing include two types of resampling approaches, robust centered log‐ratio (RCLR), cumulative sum scaling (CSS), total sum scaling (TSS), and over 10 other methods. Additionally, the integrated data transformation methods include various commonly used techniques, such as the arc sine square root transformation. When using the class, users can select algorithms based on the characteristics of each method and the type of data analysis. By default, both the input and output data for this class are microtable objects.

The trans_classifier class integrates a suite of commonly used operations for machine learning analyses based on microbial taxon abundance. Specifically, the function cal_split partitions the data into training and testing sets; cal_preProcess performs feature preprocessing; cal_feature_sel conducts feature selection; cal_train trains predictive models; and cal_predict generates predictions on the test set. Feature importance is evaluated by cal_feature_imp, with corresponding visualizations produced by plot_feature_imp. The functions cal_ROC and plot_ROC compute and visualize the Receiver Operating Characteristic (ROC) curve and the Precision‐Recall (PR) curve, respectively. Finally, cal_caretList, cal_caretList_resamples, and plot_caretList_resamples functions facilitate comparative analysis across multiple machine learning models.

Version 2.0 introduces a new class trans_metab designed for metabolite source analysis. Specifically, the function cal_match is employed to perform fuzzy matching between compound names and entries in the database; cal_origin is used for metabolite origin inference; and cal_origin_network converts the output generated by cal_origin into a metabolite‐bacteria network.

### Method comparison across different packages

**Figure 3:**
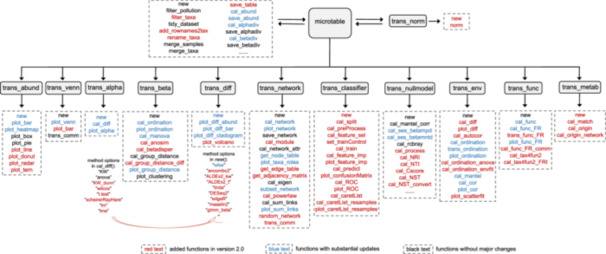
Main functions in the second‐version microeco package. The solid boxes represent classes; the dashed boxes contain the names of main functions corresponding to each class. Compared with the package version described in our previously published paper, newly added functions across all classes in the current version are marked in red; functions with substantial updates are marked in blue; the remaining functions, presented in black font, denote no major changes. In addition, the ellipsis ‘……’ indicates that other functions are not listed here. For the trans_alpha and trans_diff classes, key statistical analysis methods (option names in parameter method) are listed. Similar blue font denotes methods that have been improved, red indicates newly added methods, and black represents the original methods. The arrow from the trans_alpha class to the trans_diff class signifies that trans_diff also includes all the methods featured in trans_alpha.

Compared to its first version, the second version of the microeco package incorporates a substantial number of additional data analysis methods in each class to meet analytical needs in different scenarios. Statistical analysis is a core component of downstream omics data processing. A comprehensive summary and comparison of microbiome‐related statistical analysis methods was conducted between the microeco package 2.0 and other R packages (phyloseq, MicrobiotaProcess, and the first published version of microeco). The results indicate that the microeco package 2.0 encompasses a broader range of statistical method categories and offers more diverse analytical approaches within each category compared to other packages (Table S1). This includes both enhanced functionality at the function level (Figure 3) and extensive adjustability at the parameter level (Table S2). The parameter design takes into account different types of analytical requirements, particularly various algorithmic options and different experimental data types. Taking the trans_diff class as an example, it integrates over twenty methods for differential abundance analysis of microbiome data, with multiple operational options available for each analytical scenario (Table S3). These methods are suitable for diverse situations, such as analysis at the ASV/OTU level or higher taxonomic levels, single‐factor or multi‐factor analysis, pairwise or multi‐group comparisons, analyses with random effects or only fixed effects, and analyses involving continuous variables. Notably, the integrated Beta‐GLMM (generalized linear mixed‐effects models with beta distribution) is applicable to cases with random effects. Compared with conventional beta regression, it has a wider range of adaptability in analyzing higher‐level taxonomic abundance (e.g., genus). In the absence of random effects, Beta‐GLMM is equivalent to beta regression. Furthermore, the data access design in the current version of the trans_diff class is more standardized. Methods for analysis based on ASV/OTU abundance directly access the otu_table of the input microtable object, while methods for analysis at higher taxonomic levels access the data.frame objects within the taxa_abund list of the microtable object. For amplicon sequencing data, the data in the taxa_abund list typically consist of relative abundances, which are more suitable for analysis at higher taxonomic levels, as these methods often do not incorporate built‐in data normalization steps. Similarly, the trans_alpha class has been enhanced with multiple methods for the statistical analysis of alpha diversity, accommodating both continuous variables and the presence of random effects. For increasingly prominent categories in microbial ecology, such as machine learning models, prediction of microbial functional traits, and null models, other integrated packages do not provide related methods (Table S1). Correspondingly, the microeco package also offers a wider variety of visualization methods compared to other R packages, and these methods can be applied in more extensive contexts (Table S4). Each visualization function includes numerous parameters for customizing the graphics (Table S5), aiming to produce figures of the highest possible quality.

### Efficiency of operations and computations compared to other R packages

We compared the operational simplicity of the microeco, phyloseq, and MicrobiotaProcess packages in computing and saving relative abundances of taxa (see the code in the Supplementary Materials). Specifically, the same dataset was converted into the required input format for each package, and the identical task (computation of relative abundances across all taxonomic levels followed by result saving) was performed. The microeco package required the fewest operational steps (Figure S1). In contrast, both phyloseq and MicrobiotaProcess involved more cumbersome workflows; even when these steps were encapsulated into custom functions, they still necessitated more operations than microeco. In terms of computational time, microeco also exhibited the shortest runtime (Figure S2), demonstrating a clear overall advantage. Taking the saving of taxonomic abundance tables as an example, when exporting abundance tables for all taxonomic levels into a single merged file (e.g., in “mpa” format), microeco required fewer lines of code than phyloseq (Figure S3). In addition, the refined parameter system simplifies complex experimental analyses. For example, by introducing the by_group argument into trans_alpha and trans_beta classes, the package eliminates the need for manual data subsetting, allowing for direct statistical comparisons across sample types.

### Integration between visualization and statistical analysis

The current version of the microeco package has undergone code refactoring to improve the integration between statistical analysis and visualization. Key modifications include: (1) the decoupling of statistical analysis and visualization functions; (2) the introduction of transformation functions to enhance flexibility in visualizing complex statistical results; and (3) the comprehensive implementation of pipeline operations. Statistical analysis and visualization have been systematically separated across various classes, forming a coherent data analysis workflow. This design principle, conducting statistical analysis prior to visualization, has been consistently implemented throughout the current version. For example, in the trans_alpha class, the refactoring of functions enables plot_alpha() to automatically detect whether results from cal_diff() are present in the object, identify the statistical methods and types applied, and annotate the visualization accordingly. This refactoring effort also standardized the naming of intermediate result objects within the package. Intermediate data generated by functions are consistently prefixed with “res_” to facilitate user inspection. Similar optimizations have been applied to other classes involving the linkage between statistical analysis and visualization functions. Compared to earlier versions, the current design offers greater flexibility, maintaining workflow simplicity while allowing users to filter statistical results or save intermediate outputs locally. This approach balances simplicity and flexibility in practical user workflows, which is particularly valuable for visualizing complex statistical results. Notably, the addition of transformation functions (e.g., trans_ordination() in the trans_env class) increases adjustability prior to data visualization. The pipeline‐oriented workflow also improves maintainability in user‐defined analysis processes. Overall, the enhanced integration between visualization and statistical analysis strengthens the modular architecture of microeco, providing superior workflow construction compared to packages based on S4 classes or other functional systems.

### Method composability

In the function design of classes, certain methods are implemented by invoking methods from other classes. For example, the cal_diff function in the trans_env class performs difference testing by calling the trans_alpha class. This design approach differs somewhat from the conventional strategy of developing low‐level, general‐purpose functions. Furthermore, although different classes are partitioned into distinct analytical modules, we have paid special attention to the interface design to make it more convenient for users to combine different classes or their functions during data analysis to address specific problems (Table S6). For instance, after using the trans_func class to generate functional redundancy data, the results can be converted into the microtable class, followed by applying the trans_diff class to test the inter‐group differences across functions/traits. Similarly, after using the trans_classifier class to evaluate models and assess feature importance, users can filter specific taxa and then apply the trans_diff class to prioritize differences among key taxa. Such hierarchical data analysis is a common practice in current microbiome research.

### Advantages in parsing data with complex formats

**Figure 4:**
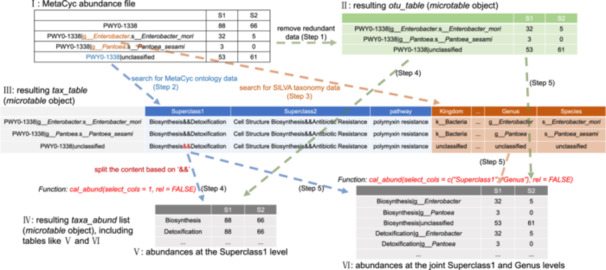
Flowchart for parsing the MetaCyc pathway abundance file with the file2meco and microeco packages. Ⅰ: MetaCyc abundance file, generated by the HUMAnN software; Ⅱ: resulting otu_table in the constructed microtable object; Ⅲ: resulting tax_table in the constructed microtable object; Ⅳ: resulting taxa_abund list in the microtable object, containing each data.frame format table (e.g., Ⅴ and Ⅵ) in this example. The step of generating the otu_table and tax_table (i.e., constructing the microtable object) is implemented by the function humann2meco from the file2meco package. In generating the otu_table (Step 1), the total abundance of metabolic pathways is excluded, as it represents the sum of all its corresponding subcategory data. Hierarchical information of each metabolic pathway is extracted from the MetaCyc ontology data (Step 2) of the file2meco package and stored in the first three columns of the resulting tax_table. If a species has abundance data associated with the given metabolic pathway, its corresponding taxonomic information is retrieved from the SILVA taxonomy table (Step 3) and saved in the tax_table data frame (starting from the Kingdom level in the 4th column). After processing the pathway abundances at the species level, the remaining pathway abundances are designated as those of unclassified species. Specifically, the row names in the otu_table are labeled as “*pathway‐name|unclassified*,” while all entries from the 4th column onward in the tax_table are marked as “*unclassified*.” If a metabolic pathway has no species‐level abundance data, all its corresponding entries are labeled as “*pathway‐name|unclassified*.” The “UNMAPPED” category is directly stored in the otu_table without further modification. Subsequent abundance calculations are performed using the function cal_abund from the microeco package. The parameter select_cols can be used to specify the columns for abundance calculation, which enables the selective combination of levels in the tax_table (e.g., the combination of metabolic pathway hierarchy and species taxonomic hierarchy). The symbol “&&” is defined as the built‐in delimiter; when detected by the function, this symbol will trigger the automatic splitting of multiple hierarchical strings (like those in Step 4). If both *Superclass1* and *Genus* are selected for abundance calculation (Step 5), the hierarchical information will be combined using “|” in the results. Note that if both *Superclass1* and *Superclass2* are selected for abundance calculation (*e.g*., selecting *Superclass1*, *Superclass2*, and *Genus*), these higher‐level classifications will not be appended to the final abundance results. This is because the multiple classification entries under these two levels (Superclass1 and Superclass2) do not necessarily exhibit a one‐to‐one correspondence.

Pathway‐level results (e.g., MetaCyc metabolic pathways) produced by the HUMAnN software integrate pathway and taxonomic information in a manner that is not readily parsable without preprocessing. To address this challenge, the humann2meco function was developed within the file2meco package to directly import raw HUMAnN output files (Figure 4). Curated microbial taxonomy information, derived from the SILVA database, has been integrated into file2meco as an internal RData object named “CHOCOPhlAn_taxonomy.” Similarly, hierarchical annotations for over 3000 MetaCyc metabolic pathways have been compiled and stored as another RData object named “MetaCyc_pathway_map.” To support multi‐label hierarchical annotations, the cal_abund function was comprehensively refactored. It now automatically parses composite labels, such as the MetaCyc pathway “Biosynthesis && Detoxification,” by recognizing specific delimiters (e.g., “&&”) and calculating abundances for each constituent category independently (see Step 4 of Figure 4). The enhanced cal_abund function in microtable class automatically recognizes the delimiter “&&” between multiple labels, splits the composite annotation accordingly, and computes abundances for each constituent category separately. Furthermore, the addition of the select_cols parameter enables users to flexibly specify which taxonomic or pathway hierarchy levels to include in abundance calculations (see Supplementary Code). When relative abundances are not required, the rel argument can be set to FALSE to retain absolute values. This enhanced flexibility significantly broadens the range of data types that microeco can process, thereby maximizing the utility of its existing analytical framework. Consequently, the synergistic integration of file2meco and microeco provides users with a comprehensive, end‐to‐end workflow for downstream analysis of complex microbiome datasets.

### A workflow example

**Figure 5:**
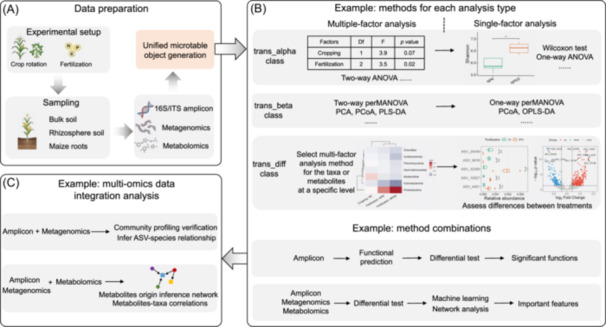
Schematic diagram of an exemplary analysis workflow based on actual experimental data. (A) Experimental design, sampling, omics measurement, and data preprocessing. 16S/ITS amplicon: 16S rRNA gene and ITS fragment amplicon sequencing; (B) Analysis example based on a single type of omics data, consisting of two parts: the upper section shows the operations under each type of analysis method according to the experimental design, and the lower section presents the analyses under different combinations of analysis methods. Relevant code for each section is available in the GitHub repository (https://github.com/ChiLiubio/microeco_protocol_v1). The *p*‐values shown for the “trans_diff” category have all been adjusted using the Benjamini–Hochberg false discovery rate (FDR) approach. (C) Example of multi‐omics integrative analysis. Relevant code is available in the GitHub repository (https://github.com/ChiLiubio/microeco_protocol_v1), where we have added analyses such as the “Infer ASV‐species relationship” (in Step 26) and “Metabolites origin inference network” (in Step 31) for multi‐omics integrative studies. Users can also refer to the online tutorial (https://chiliubio.github.io/microeco_tutorial/) for other examples. *p < 0.05; **p < 0.01; ***p < 0.001. perMANOVA, permutational multivariate analysis of variance; OPLS‐DA, orthogonal partial least squares discriminant analysis; PCA, principal component analysis; PCoA, principal coordinates analysis; PLS‐DA, partial least squares discriminant analysis.

We used a multi‐omics dataset with multi‐factor treatments to demonstrate a workflow example (Figure 5). The omics data included amplicon sequencing of 16S rRNA genes and internal transcribed spacer (ITS) fragments from bulk soil, rhizosphere soil, and maize root endosphere, metagenomic sequencing of bulk soil and rhizosphere soil, as well as untargeted metabolomic data. To highlight the advantages of the second version, we no longer focus on the selection of specific methods, but instead emphasize the transition from multi‐factor analysis to single‐factor or specific group comparisons, the importance of combining methods from different categories in the package during analysis, and how different types of omics data can be jointly analyzed. Detailed usable code workflows are described in the legend of Figure 5.

## DISCUSSION

### Necessity of methodological diversification

The second version of microeco package has achieved substantial improvements in the integration and development of analytical methods. Different methods belonging to the same category may vary in their scope of applicability, thus necessitating targeted selection during practical implementation. This constitutes the primary rationale for the integration of a comprehensive suite of ecological and statistical methods into the functional framework of the package. Distinct methods may yield disparate outcomes when addressing a specific research question. For instance, Nearing et al. selected 38 16S rRNA gene datasets and applied 14 differential taxa analysis methods to evaluate their performance [15]. The results revealed that the number of significant taxa detected by most methods was closely correlated with data preprocessing protocols, sample size, sequencing depth, and the effect size of community differences. From this perspective, the diverse differential test methods incorporated into the trans_diff class of the microeco package facilitate users in comparing the performance of different methods and selecting the optimal one tailored to specific data analysis scenarios [16]. Currently, different research directions tend to exhibit distinct methodological preferences, leading to discrepancies in the methods of interest among researchers from diverse domains. Therefore, a high degree of methodological diversification can broaden the scope of application and enable users to rapidly access and implement novel methods that may be more suitable for their specific research questions.

The microeco package has been widely utilized and included in methodological comparisons alongside other R packages [2], which indirectly reflects the strong demand among users for R packages characterized by methodological diversification. Beyond statistical analysis, the diversification of visualization approaches not only allows for flexible integration with statistical results but also caters to different experimental design types and data analysis requirements. For example, the genus‐level relative abundances fitted by the Beta‐GLMM method implemented in the microeco package, together with heatmap visualization of the results, have intuitively revealed the effects of crop rotation and fertilization regimes on the abundances of several key bacterial, fungal, and viral genera [17]. Concurrently, these heatmaps enable convenient identification of the abundance trends of these genera in the rhizosphere and endosphere compared with those in the bulk soil. Hence, for multi‐factor experimental designs, heatmap visualization offers greater information density. Through multi‐factor visualization, researchers can rapidly screen for important taxa or influencing factors, and subsequently employ other methods to conduct group‐specific visualization of these target taxa, thereby establishing a rational data analysis workflow. This approach also proves highly efficient for single‐factor, multi‐group experiments aimed at investigating the variation trends of different taxa across various groups relative to the control group. A typical example is the comparison of microbial community variation trends across multiple plant compartments relative to bulk soil, as well as the identification of consistent variation patterns among different compartments [18]. In conclusion, methodological diversification holds significant value for enhancing the efficiency of data analysis for end users.

### Unique features and advantages of the package architecture design

The current microeco package places great emphasis on the integration between statistical analysis and visualization. Many contemporary R packages for data analysis incorporate statistical methods directly into their visualization functions, often by designing statistical approaches as adjustable parameters. While this design paradigm is indeed suitable for relatively straightforward visualization techniques and statistical analyses, it may prove limiting when experimental designs grow in complexity. In such scenarios, users often need to experiment with different visualization methods and integrate results from diverse types of statistical analyses. Furthermore, many statistical methods in microbiome data analysis are inherently complex. Their results frequently require filtering and refinement before being suitable for visualization. The flexibility to incorporate such user‐defined, iterative operations into a streamlined analytical workflow has long been overlooked. The primary challenge lies in implementing this flexibility within a modular design framework. The microeco package version 2 addresses this by leveraging R6 classes (where both data and functions are treated as objects). Building on this, the refinement of internal functions and structural design ensures a superior user experience compared to S4‐based packages such as phyloseq and MicrobiotaProcess (as demonstrated in Figures S1 and S3). It is also noteworthy that specially designed transformation functions, which convert complex analytical results into plottable formats, represent an extension of the core philosophy of separating statistical computation from visualization steps. This strategy allows users to more clearly understand the analytical design, thereby facilitating the extraction of transformed data. This benefit becomes particularly evident when users need to inspect or export data for downstream analysis with other tools.

In microeco version 2, the modular, compositional development paradigm reduces code redundancy in the implementation of modular design and enhances the maintainability of the package. From the end‐user perspective, as illustrated in the Results section, when a single class is insufficient to address complex analytical tasks, users can integrate different classes to accomplish comprehensive data analyses. This flexible compositional capability is also embodied in the extended packages developed based on microeco, such as meconetcomp [19] and mecoturn [20]. Many methods within these two packages were developed by integrating and extending the methodologies of microeco to address specific challenges in network comparison and community turnover analysis. This class‐level composability significantly elevates the application value of the microeco package. Thus, the formal segregation of code into distinct classes does not result in operational isolation for users. Once they clarify the core analytical objectives targeted by each class, users can more readily implement joint applications of multiple classes for customized analyses.

In terms of workflow design philosophy, the microeco package focuses on the conciseness and flexibility of structure design compared with other R packages. For the workflow combination and execution, the microeco 2 enables pipelining of functions within a class using the $ operator, analogous to conventional pipe operators. This pipelining mechanism offers high adjustability for users: if result filtering is required prior to visualization, users can first assign the analytical object to an intermediate variable, followed by standalone visualization after data filtration. Conversely, if no filtration is needed, visualization functions can be directly integrated into the pipeline along with statistical analysis procedures. For relatively mature omics technologies, streamlined workflows are valuable for a broad user base, as it facilitates rapid data analysis and enables researchers to obtain valid results with minimal time investment. Therefore, the workflow‐driven operational paradigm implemented in the microeco 2 exhibits strong compatibility and high practical utility. Moving forward, we plan to develop an interactive analytical tool built upon the Shiny package, designed to help beginners learn and apply the microeco package for omics analyses efficiently.

## CONCLUSION

In summary, the microeco package version 2.0 represents a substantial update compared to its initial release, improving both design coherence and methodological coverage. Together with its extension packages, microeco offers a robust and integrated downstream analytical framework for omics data across diverse areas of microbiome research.

## METHODS

### Primary development efforts for version 2.0 of the microeco package

#### Development of new classes

Amplicon sequencing data often require normalization for downstream analysis. For instance, sequencing depth must be considered in diversity analyses, while compositionality and sparsity need to be addressed in some differential abundance analyses [21]. In the current version, a new trans_norm class has been introduced, dedicated to performing various normalization methods, particularly those tailored for amplicon sequencing data. The integrated methods include: Two rarefaction‐based approaches: the commonly used rarefying [22] and the method from the SRS package [23]; Centered log‐ratio (CLR) [24]; RCLR [25]; CSS [26]; Geometric mean of pairwise ratios (GMPR) [27]; Wrench [28]; Relative log expression (RLE) and total sum scaling (TSS, also referred to as Proportion). These developments were achieved by integrating existing R packages, publicly available code for specific methods, and custom implementations. Furthermore, additional data transformation methods have been added, based on the decostand function from the vegan package [6] as well as custom code.

Machine learning methodologies have been increasingly adopted in microbial ecology due to their ability to model complex, non‐linear relationships in high‐dimensional microbiome datasets without relying on strict parametric assumptions [29]. A novel class, trans_classifier, has been specifically implemented to facilitate machine learning‐related analyses. It constructs predictive models based on taxonomic abundances, with individual functions designed in alignment with common analytical procedures and objectives. The majority of functions and methods within this class are developed based on the caret package [30]. In addition, a feature selection function has been designed based on the Boruta algorithm [31]. Functions for plotting ROC curves and PR curves have been developed leveraging the multiROC package [32]. For the calculation of feature significance in random forest analyses, the rfPermute package [33] is employed.

#### Integration of diverse algorithms

More methods have been integrated into the existing classes and functions to serve a wider range of analytical purposes. In the cal_diff function of the trans_alpha class, several methods, including linear regression, have been incorporated. Among these, Dunn's Kruskal–Wallis Multiple Comparisons method was developed based on the FSA package [34], and the mixed‐effects models are computed using the lmerTest package [35]. The trans_beta and trans_env classes have been expanded with detrended correspondence analysis and correspondence analysis (CCA) methods, respectively, implemented via the vegan package. Within the trans_diff class, multiple differential analysis methods have been added, including ALDEx2 [36], ANCOM‐BC2 [37], and linda [38]. Additionally, a Beta‐GLMM was developed based on the glmmTMB package [39] for fitting the relative abundance (ranging from 0 to 1) of taxa at higher taxonomic levels (e.g., genus, phylum) in the presence of random effects. To ensure the data strictly lies between 0 and 1, a small constant was added to zero abundances, and values of 1 were constrained to be less than 1 using the transformation 1/(1 + small constant). A detailed description of the Beta‐GLMM model is provided in the Supplementary Material. Furthermore, the statistical analysis methods from the trans_alpha class have also been integrated into the trans_diff class to facilitate common statistical analyses, along with the addition of data transformation methods. In the cal_network function of the trans_network class, the BEEM‐Static network construction method [40] has been incorporated. Finally, in the cal_func function of the trans_func class, two databases have been added: the FungalTraits database [41] for predicting fungal functional traits and the NJC19 database [42] for predicting bacterial metabolic traits.

#### Optimization of the function and parameter architecture

The functions and parameters of existing classes were optimized to broaden the applicability of the current class system. This primarily includes: (1) Adding general‐purpose functions. In the trans_abund class, multiple functions such as plot_line and plot_donut have been added to generate line charts and donut charts. In the trans_venn class, the plot_bar function has been added to create UpSet plots. In the trans_beta class, the cal_anosim function has been incorporated to implement analysis of similarities (ANOSIM). Additionally, the cal_group_distance_diff function has been introduced to perform statistical analysis on distance transformation results. In the trans_diff class, the plot_volcano function has been added to generate volcano plots. To enhance the flexibility of visualizing complex statistical analysis results, several transformation functions have been integrated, such as the trans_ordination function in the trans_env class and the trans_func_FR function in the trans_func class. Within the trans_nullmodel class, functions cal_NST and cal_Cscore have been added to calculate normalized stochasticity ratio (NST) [43] and checkerboard score (C score), respectively; functions cal_NRI and cal_NTI have also been added to compute the net relatedness index (NRI) and nearest taxon index (NTI) metrics, respectively. In the trans_network class, cal_module has been added to perform modularity calculations in a more flexible manner; cal_powerlaw has also been incorporated to facilitate the fitting of node degree to a power‐law distribution. In the trans_func class, the functions cal_tax4fun2 and cal_tax4fun2_FRI have been added to implement the Tax4Fun2 method [44]. Within these functions, the original methodological framework has been optimized to enable more efficient computation and to output the functional profiles of reference genomes matched by ASVs/OTUs representative sequences. The cal_func_FR_comm function has been introduced to compute functional redundancy (FR) at the community level, based on the FR values of individual functions generated by the cal_func_FR function. This is defined as the geometric mean of FR for each function/trait within a community:

$$FR_{k}=\sqrt[{n}]{FR_{k1}\times FR_{k2}\times \cdots \times FR_{\mathrm{kn}}},$$

where $FR_{k}$ denotes the FR at the community level for sample k. $FR_{\mathrm{kn}}$ represents the FR of function n for sample k. (2) Incorporating key parameters. The parameters of the cal_manova function in the trans_beta class have been restructured to enable the function to perform overall multi‐factor or single‐factor permutational multivariate analysis of variance (perMANOVA) tests, as well as pairwise comparisons. These operations can be conducted separately across different groups via the by_group parameter. The new function in the trans_alpha class has been augmented with a by_group parameter to facilitate subsequent statistical analyses within subgroups. In the cal_cor function of the trans_env class, a partial parameter has been added to control whether partial correlation analysis should be employed based on the ppcor package [45]. In the cal_func_FR function of the trans_func class, the parameter adj_tax has been introduced to represent an adjustment factor (AF) for extending the FR calculation method. This parameter quantifies the dispersion of functional groups across taxonomic lineages, with a default value of FALSE. The AF ranges from 0 to 1; higher values indicate greater taxonomic dispersion (at selectable taxonomic levels) of ASVs/OTUs possessing a specific functional trait, thereby implying higher functional redundancy. (3) Adjusting function naming conventions. In trans_func, the cal_spe_func function has been renamed to cal_func, and cal_spe_func_perc has been renamed to cal_func_FR to better reflect FR. In trans_env, the cal_rda function has been renamed to cal_ordination to facilitate easier methodological extensions. (4) Refactoring code within certain class functions to strengthen the integration between statistical analysis results and visualization. (5) Extending capabilities to multi‐omics approaches. The new version includes several methods commonly used in transcriptomics and metabolomics data analysis. For instance, the DESeq2 [46] and edgeR [47] methods in the trans_diff class can be applied for differential abundance analysis in transcriptomics/metatranscriptomics data, while the maaslin3 [48] method is suitable for meta‐omic association discovery. The cal_ordination function in the trans_beta class has been extended to incorporate partial least squares discriminant analysis (PLS‐DA) and orthogonal PLS‐DA (OPLS‐DA) [49] methods for metabolomics data analysis. In version 2.1.0, we introduced the trans_metab class specifically designed for the source analysis of metabolites within metabolomics data. This class was developed based on the data structures provided by the tidymass2 package [50]. Other general‐purpose methods available in classes such as trans_abund, trans_venn, trans_classifier, and trans_env are also applicable to the analysis of these omics datasets.

#### Parsing complexly formatted data

Bioinformatic analysis results of sequencing data, such as metagenomics and metatranscriptomics, typically exhibit sophisticated data formats. For instance, the gene or metabolic pathway abundance data generated by the HUMAnN software [13] also incorporates taxonomic information across different species. This necessitates flexible selection for the subsequent calculation of relative abundances or RPK abundances of genes/metabolic pathways at various hierarchical levels. In addition, the ontology information within the MetaCyc database [14] may be annotated with multiple labels at the superclass level, which also requires the calculation of metabolic pathway abundances to accommodate such complex data formats. Similarly, certain nitrogen cycling genes included in the NCycDB database [51] may be assigned to multiple categories; for example, the *narG* gene belongs to both the “Denitrification” and “Dissimilatory nitrate reduction” categories. Accordingly, the microtable class of the microeco package has been refined to accommodate these characteristics, enabling it to accept user‐input hierarchical information (e.g., combinations of metabolic pathway and taxonomic hierarchies) and address the issue of multi‐label annotations. The solution for processing multi‐label information involves splitting the input labels by the delimiter and performing separate calculations for each label during abundance quantification. Methods for file reading and parsing have been integrated into the file2meco package [52], allowing the microeco package to focus exclusively on downstream data analysis operations. Within the file2meco package, the function humann2meco has been specifically designed to read metabolic pathway result files generated by the HUMAnN software. The development workflow of this function is as follows: (1) First, a taxonomic classification table (in data.frame format) covering prokaryotic taxa from the kingdom to genus level was compiled based on the SILVA database. (2) Based on the MetaCyc database (https://metacyc.org/), the “Ontology” information for each metabolic pathway (3241 pathways in total as of October 2024, based on the pathway IDs) was manually retrieved. The highest‐level category was designated as “Superclass1,” and the next‐level category as “Superclass2.” For entries with multiple ontology annotations, the relevant labels were merged using the specific delimiter “&&.” For metabolic pathways classified as superpathways, only those uniquely belonging to this category were annotated as “Superpathways.” Ultimately, a hierarchical information table for metabolic pathways was constructed, encompassing three levels: “Superclass1,” “Superclass2,” and “pathway.”

### Comparative analysis with other R packages

The amplicon sequencing dataset used for methodological evaluation was obtained from a published study [16]. The analyses were conducted using the following R package versions: phyloseq [8] v1.50.0, MicrobiotaProcess [10] v1.18.0, and microeco v2.0.0. The code employed for taxonomic abundance calculation is provided in the supplementary materials. All computations were performed in R version 4.4.1 on a Windows 10 operating system, with platform architecture x86_64‐w64‐mingw32/x64 (64‐bit). The hardware configuration included an Intel(R) Core(TM) i7‐4710MQ CPU running at 2.50 GHz, and all analyses were executed in single‐threaded mode. Each method was repeated 10 times, and the mean value was reported. To comparatively assess the extent to which each R package integrates diverse analytical approaches, we quantified either (i) the number of distinct methods of a given category implemented within a package, or (ii) the degree of parameter configurability available for a specific method. Results are represented by the number of plus signs (“+”): a higher number of “+” symbols indicates greater availability of relevant functions, methodological options, or adjustable parameters within the package, thereby reflecting a broader applicability across diverse microbiome data analysis scenarios. Packages lacking implementation of a particular method were left unmarked.

## AUTHOR CONTRIBUTIONS

**Chi Liu**: Conceptualization; methodology; software; validation; writing—original draft; writing—review and editing; data curation. **Xiangzhen Li**: Conceptualization; methodology; supervision; resources; writing—review and editing; funding acquisition. **Felipe R. P. Mansoldo**: Methodology; Software; validation; writing—original draft. **Tong Chen**: Validation; writing—review and editing. **Fanzheng Meng**: Validation; writing—review and editing. **Ruixiang Tang**: Validation; writing—review and editing. **Siyu Zhou**: Validation; writing—review and editing. **Qinghua Yang**: Validation; writing—review and editing; supervision. **Ruixin Shao**: Validation; writing—review and editing; supervision; funding acquisition. **Minjie Yao**: Conceptualization; methodology; writing—review and editing; funding acquisition; supervision; resources. All authors have read the final manuscript and approved it for publication.

## CONFLICT OF INTEREST STATEMENT

The authors declare no conflicts of interest.

## ETHICS STATEMENT

No animals or humans were involved in this study.

## Supporting information


**Figure S1:** Comparison of lines of code needed for relative abundance analysis using various R packages.
**Figure S2:** Comparison of relative abundance calculation time using different R packages.
**Figure S3:** Lines of code required to merge and save relative abundance in MPA format across R packages.

**Table S1:** Comparison of statistical method coverage across different R packages.
**Table S2:** Number of integrated methods in some statistical analysis functions.
**Table S3:** Summary of the number of available methods under different scenarios in differential abundance analysis.
**Table S4:** Comparison of visualization method coverage across different R packages.
**Table S5:** Summary of the number of parameters for some visualization functions.
**Table S6:** Examples of composability for each class.

## Data Availability

The code of microeco package is deposited in GitHub repository (https://github.com/ChiLiubio/microeco) and Gitee repository (https://gitee.com/chiliubio/microeco). The reference analytical workflow released in sync with the microeco package update includes an online tutorial (https://chiliubio.github.io/microeco_tutorial) and a published protocol (https://github.com/ChiLiubio/microeco_protocol_v1). Supplementary materials (figures, tables, graphical abstract, slides, videos, Chinese translated version, and updated materials) may be found in the online DOI or iMeta Science (http://www.imeta.science). The data that support the findings of this study are openly available in GitHub repository (https://github.com/ChiLiubio/microeco_protocol_v1).
