# Supplementary material for: microeco 2: A comprehensive R package for downstream analysis of microbiome omics data
Source: Imeta. 2026 May 16;5(3):e70132. doi: 10.1002/imt2.70132 (PMC13377413; doi:10.1002/imt2.70132)
Supplement: Supplementary file 1 — Figure S1: Comparison of lines of code needed for relative abundance analysis using various R packages. Figure S2: Comparison of relative abundance calculation time using different R packages. Figure S3: Lines of code required to merge and save relative abundance in MPA format across R packages. [file IMT2-5-e70132-s002.docx]

**Supporting Information to**

**microeco 2: A comprehensive R package for downstream analysis of microbiome omics data**

**Running title:** Second version of R microeco package

Chi Liu ^1, 2^, Xiangzhen Li ^1*^, Felipe R. P. Mansoldo ^3^, Tong Chen ^4^, Fanzheng Meng ^2^, Ruixiang Tang ^5^, Siyu Zhou ^6^, Qinghua Yang ^2^, Ruixin Shao ^2*^, Minjie Yao ^1*^

^1^Engineering Research Center of Soil Remediation of Fujian Province University; College of Resources and Environment, Fujian Agriculture and Forestry University, Fuzhou 350002, China

^2^State Key Laboratory of High-Efficiency Production of Wheat-Maize Double Cropping/College of Agronomy, Henan Agricultural University, Zhengzhou 450046, China

^3^Universidade Federal do Rio de Janeiro, Instituto de Química, LAGOA-LADETEC, Rio de Janeiro, Rio de Janeiro 21941-909, Brazil

^4^State Key Laboratory for Quality Ensurance and Sustainable Use of Dao‐di Herbs, National Resource Center for Chinese Materia Medica, China Academy of Chinese Medical Sciences, Beijing 100000, China

^5^Key Laboratory of Bioresources and Ecoenvironment (Ministry of Education), Sichuan Key Laboratory of Conservation Biology on Endangered Wildlife, College of Life Sciences, Sichuan University, Chengdu 610065, China

^6^State Key Laboratory of Genetic Engineering, School of Life Sciences, Human Phenome Institute, Fudan University, Shanghai 200433, China

*Correspondence: [lixz@fafu.edu.cn](mailto:lixz@cib.ac.cn) (Xiangzhen Li); [shao_rui_xin@126.com](mailto:shao_rui_xin@126.com) (Ruixin Shao); [yaomj@fafu.edu.cn](mailto:yaomj@fafu.edu.cn) (Minjie Yao)

**Supplementary codes**

#### R Code related to taxonomic abundance calculation and file saving#####

########################### R Code Part 1##########################

###### Taxonomic abundance calculation at each taxonomic level across different packages ######

####################################R Code#########################################

# Prepare the required data for the microeco package (v2.0.0)
library(microeco)
data(dataset)
meco <- clone(dataset)
# Prepare the required data for the phyloseq package (v1.2.1)
library(file2meco)
library(phyloseq)
physeq <- meco2phyloseq(dataset)
# Prepare the required data for the MicrobiotaProcess package (v1.18.0)
# Perform conversion based on the ‘physeq’ object
library(MicrobiotaProcess)
mpse <- as.MPSE(physeq)

#################################################################################

# First, demonstrate the calculation of the relative abundance of taxa at each level

# using the microeco package, and save the results to a folder.

####################################for microeco ##################################

# Calculate the relative abundance of each taxon
meco$cal_abund(rel = TRUE)
# Save the relative abundance to a local file
meco$save_abund(dirpath = "meco_abund")

#################################################################################

# Calculate and save the relative abundance of taxa at each level using the phyloseq package.

##################################for phyloseq####################################

# Calculate the relative abundance of each taxon
physeq_abund <- list()
for(i in 1:length(rank_names(physeq))) {
 taxrank = rank_names(physeq)[i]
 glom1 <- tax_glom(physeq, taxrank = taxrank)
 glom_table <- as.data.frame(glom1@otu_table@.Data)
 glom_table[] <- lapply(glom_table, function(x){x/sum(x)})
 rownames(glom_table) <- apply(glom1@tax_table@.Data[, 1:i, drop = FALSE], 1, paste0, collapse = "|")
 physeq_abund[[taxrank]] <- glom_table
}
# Save the relative abundance to a local file
dir.create("physeq_abund")
for(j in names(physeq_abund)){
 write.csv(physeq_abund[[j]], paste0("physeq_abund/", j, "_abund.csv"))
}

#################################################################################

# Then, calculate and save the relative abundance of taxa at each level

# using the MicrobiotaProcess package.

##############################for MicrobiotaProcess ################################

# Calculate the relative abundance of each taxon
mpse %<>% mp_cal_abundance(.abundance = Abundance, force = TRUE)
mpse_abund <- list()
for(i in c("Kingdom", "Phylum", "Class", "Order", "Family", "Genus", "Species")){
 tmp <- mp_extract_abundance(mpse, taxa.class = !!i)
 tmp %<>% tidyr::unnest(cols = AbundanceBySample)
 abund <- reshape2::dcast(tmp, label ~ Sample, value.var = "RelAbundanceBySample")
 rownames(abund) <- abund[, 1]
 abund <- abund[, -1] %>% {./100}
 abund <- abund[, mpse$SampleID]
 mpse_abund[[i]] <- abund
}
# Save the relative abundance to a local file
dir.create("mpse_abund")
for(j in names(mpse_abund)){
 write.csv(mpse_abund[[j]], paste0("mpse_abund/", j, "_abund.csv"))
}

##############################################################################

############################# R Code Part 2#################################

### Saving taxonomic abundances to a local file with the ‘mpa’ format for different packages ###

# Continuing from the results of the previous section-Part 1
# Save the relative abundance of the meco object as a single file in mpa format
meco$save_abund(dirpath = "meco_abund_merge", merge_all = TRUE, rm_un = TRUE)

# Continuing from the results of the previous section-Part 1
# Save the relative abundance of the physeq_abund object as a single file in mpa format
all_table <- do.call(rbind, physeq_abund)
all_table <- data.frame(Taxa = rownames(all_table), all_table)
all_table <- all_table[!grepl("__$", all_table$Taxa), ]
write.table(all_table, file = "physeq_abund_merge.tsv", row.names = FALSE, sep = "\t")

##############################################################################

######################### R Code Part 3########################

########### Analysis examples using the result from HUMAnN software##########

####################################R Code#########################################

# Load file2meco package and microeco package
library(file2meco)
library(microeco)
# Use the MetaCyc metabolic pathway abundance data built into the file2meco package
# Obtain the path of the required file
sample_file_path <- system.file("extdata", "example_metagenome_sample_info.tsv", package="file2meco")
match_file_path <- system.file("extdata", "example_metagenome_match_table.tsv", package="file2meco")
abund_file_path <- system.file("extdata", "example_HUMAnN_MetaCyc_abund.tsv", package="file2meco")

# Read the abundances generated by the HUMAnN software.
test <- humann2meco(abund_file_path, db = "MetaCyc", sample_table = sample_file_path, match_table = match_file_path)

# Enrichment calculation of each metabolic pathway
# rel = FALSE: based on raw RPK data instead of relative abundance
test$cal_abund(select_cols = 1:3, rel = FALSE)
# Save the results to a local file
test$save_abund(dirpath = "MetaCyc_pathways_RPK")
# Enrichment calculation of the taxa to which metabolic pathways belong, from the kingdom to genus levels
test$cal_abund(select_cols = 4:9, rel = TRUE)
# Save the results to a local file
test$save_abund(dirpath = "MetaCyc_taxa_rel")
# Combined information of metabolic pathways and taxonomy (Kingdom level)
test$cal_abund(select_cols = c(1, 4), rel = FALSE)
# Save the results to a local file
test$save_abund(dirpath = "MetaCyc_pathandtaxa_RPK")

#####################################################################################

**METHODS**

**Details of Beta-GLMM (generalized linear mixed-effects models with the beta distribution as family function)**

For relative abundance data $y_{ij}\in\left( 0,1 \right)$ (where $i=1,...,N$ denotes groups or subjects, and $j=1,...,n_{i}$ denotes observations within a group), the Beta-GLMM can be expressed as:

$$g\left( \mu_{ij} \right)=\mathbf{X}_{ij}\boldsymbol{\beta}+\mathbf{Z}_{ij}\mathbf{b}_{i}$$

where: $\mu_{ij}=E\left( y_{ij}\mid\mathbf{b}_{i} \right)$ is the conditional mean given the random effects; $g\left( \cdot\right)$ is the link function, adopting the logit link: $g\left( \mu_{ij} \right)=log\left( \frac{\mu_{ij}}{1-\mu_{ij}} \right)$; $\mathbf{X}_{ij}$ is the design matrix for fixed effects; $\boldsymbol{\beta}$ is the parameter vector for fixed effects; $\mathbf{Z}_{ij}$ is the design matrix for random effects; $\mathbf{b}_{i}\sim N\left( \mathbf{0},\boldsymbol{\Sigma}_{b} \right)$ is the random effects vector, with $\boldsymbol{\Sigma}_{b}$ representing the covariance matrix of random effects.

The response variable $y_{ij}$ follows a conditional Beta distribution:

$$y_{ij}\mid\mathbf{b}_{i}\sim\text{Beta}\left( \mu_{ij}\phi,\left( 1-\mu_{ij} \right)\phi\right)$$

Its probability density function is:

$$f\left( y_{ij}\mid\mu_{ij},\phi\right)=\frac{\Gamma\left( \phi\right)}{\Gamma\left( \mu_{ij}\phi\right)\Gamma\left( \left( 1-\mu_{ij} \right)\phi\right)}y_{ij}^{\mu_{ij}\phi-1}\left( 1-y_{ij} \right)^{\left( 1-\mu_{ij} \right)\phi-1}$$

where $\phi>0$ is the precision parameter, and the conditional variance structure is:

$$\text{Var}\left( y_{ij}\mid\mathbf{b}_{i} \right)=\frac{\mu_{ij}\left( 1-\mu_{ij} \right)}{1+\phi}$$

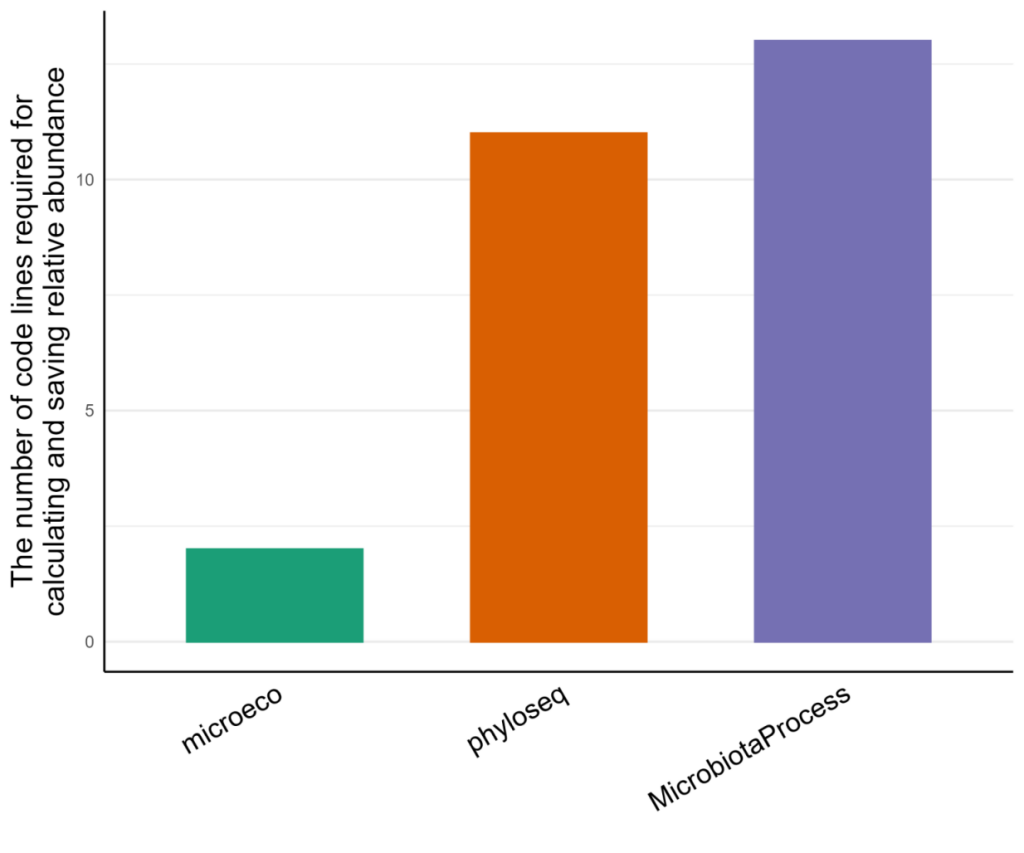


**Figure S1 Comparison of lines of code needed for relative abundance analysis using various R packages.**


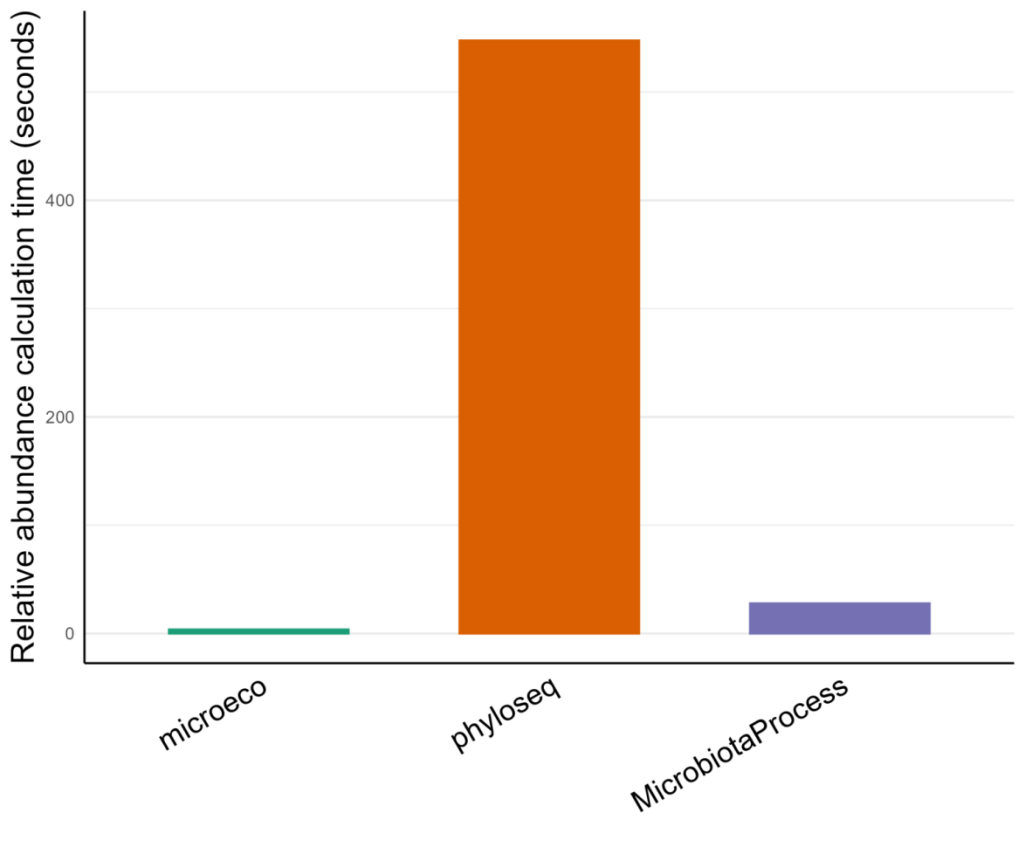


**Figure S2 Comparison of relative abundance calculation time using different R packages.**


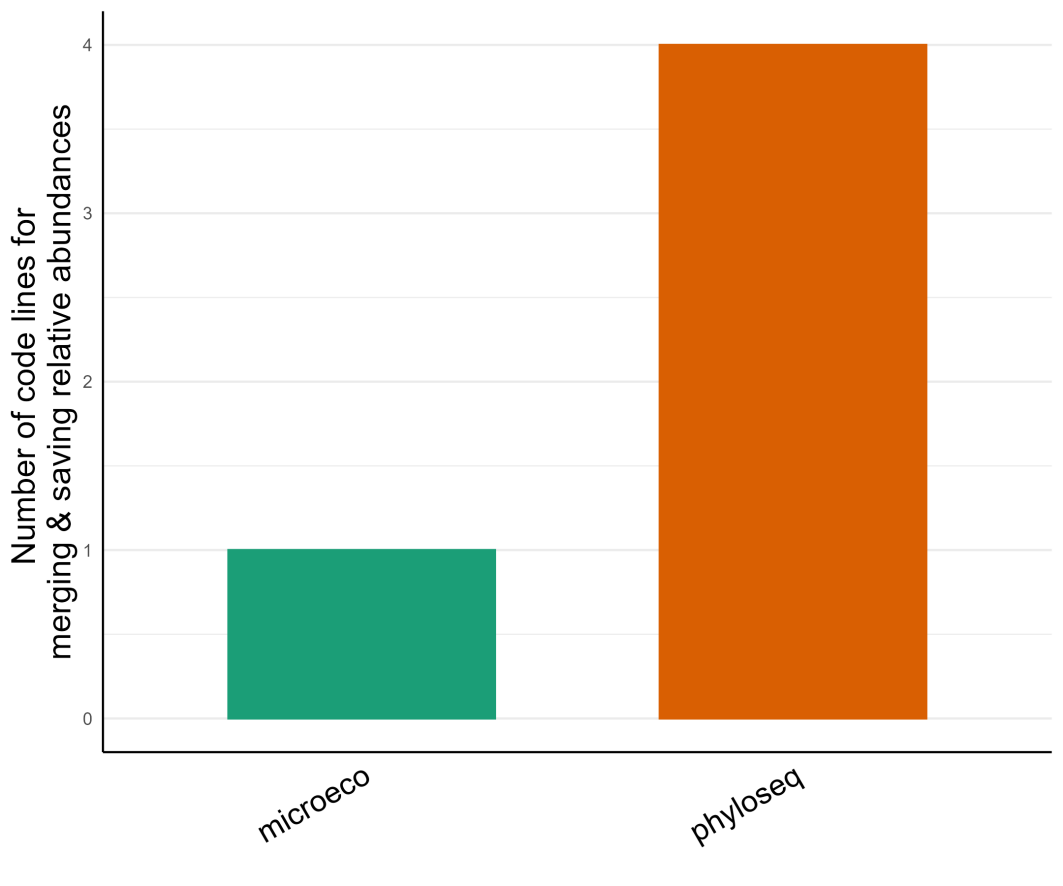


**Figure S3 Lines of code required to merge and save relative abundance in MPA format across R packages.**
